# A pharmaco-economic analysis of patients with schizophrenia switching to generic risperidone involving a possible compliance loss

**DOI:** 10.1186/1472-6963-9-32

**Published:** 2009-02-18

**Authors:** Maarten Treur, Bart Heeg, Hans-Jürgen Möller, Annette Schmeding, Ben van Hout

**Affiliations:** 1 Pharmerit BV, Rotterdam, The Netherlands; 2 Psychiatrische Klinik der Ludwig-Maximilians-Universität München, München, Germany; 3 Janssen-Cilag GmbH, Raiffeisenstrasse 8, 41470 Neuss, Germany

## Abstract

**Background:**

As schizophrenia patients are typically suspicious of, or are hostile to changes they may be reluctant to accept generic substitution, possibly affecting compliance. This may counteract drug costs savings due to less symptom control and increased hospitalization risk. Although compliance losses following generic substitution have not been quantified so far, one can estimate the possible health-economic consequences. The current study aims to do so by considering the case of risperidone in Germany.

**Methods:**

An existing DES model was adapted to compare staying on branded risperidone with generic substitution. Differences include the probability of non-compliance and medication costs. Incremental probability of non-compliance after generic substitution was varied between 2.5% and 10%, while generic medication costs were assumed to be 40% lower. Effect of medication price was assessed as well as the effect of applying compliance losses to all treatment settings. The probability of staying on branded risperidone being cost-effective was calculated for various outcomes of a hypothetical study that would investigate non-compliance following generic substitution of risperidone.

**Results:**

If the incremental probability of non-compliance after generic substitution is 2.5%, 5.0%, 7.5% and 10% respectively, incremental effects of staying on branded risperidone are 0.004, 0.007, 0.011 and 0.015 Quality Adjusted Life Years (QALYs). Incremental costs are €757, €343, -€123 and -€554 respectively. Benefits of staying on branded risperidone include improved symptom control and fewer hospitalizations. If generic substitution results in a 5.2% higher probability of non-compliance, the model predicts staying on branded risperidone to be cost-effective (NICE threshold of ₤30,000 per QALY gained). Compliance losses of more than 6.9% makes branded risperidone the dominant alternative. Results are sensitive to the locations at which compliance loss is applied and the price of generic risperidone. The probability that staying on branded risperidone is cost-effective would increase with larger compliance differences and more patients included in the hypothetical study.

**Conclusion:**

The model predicts that it is cost-effective to keep a patient with schizophrenia in Germany on branded risperidone instead of switching him/her to generic risperidone (assuming a 40% reduction in medication costs), if the incremental probability of becoming non-compliant after generic substitution exceeds 5.2%.

## Background

Compliance amongst patients with schizophrenia is an important issue. Reported non-compliance rates for patients suffering from schizophrenia are relatively high, ranging from 40% to 50% [1-5] Non-compliance has been found to significantly increase the likelihood of hospitalization, inpatient charges, switching or augmenting therapy and symptoms.[1,6,7] Factors influencing compliance behaviour of patients with schizophrenia can be classified into patient-related (e.g. nature of the illness and attitude towards the illness), environment-related (e.g. social support and therapeutic environment), physician-related (e.g. clinician-patient relation and information) and treatment-related (e.g. side effects and route of administration).[8]

With regard to treatment related factors, poor compliance can result from generic substitution; patients might be reluctant to accept a switch to a generic version due to differences in colorants, size, shape or even name.[9,10] Ganther *et al*. showed that generic alternatives are also thought to be less safe and less effective than their branded equivalents by at least 20% to 30% of consumers.[11] To some extent this applies to all medications, however the specific nature of schizophrenia aggravates poor compliance. Patients suffering from schizophrenia are suspicious to change in general and often develop paranoia and suffer from delusions. Some authors have even noted that when patients are stable on a certain medication for schizophrenia they may not readily accept a sudden switch to a generic version and may even regard it as a poisoning attempt.[12] In such situations it may be cost-saving/cost-effective in the long-term to maintain a stable regimen. Generic substitution immediately saves drug costs but, when compliance is negatively affected, this can be outweighed by poorer symptom control and increased hospitalization costs. According to an international review of the costs of schizophrenia, a modest 1 to 9% of all direct health care costs in schizophrenia are attributed to drug costs, whereas one to two-thirds are attributed to hospitalization.[13]

This report quantifies the health-economic impact of generic substitution involving a possible compliance loss, by considering the case of oral risperidone in Germany (for which the patent expired in December 2007) using a pharmaco-economic Discrete Event Simulation (DES) model.

## Methods

An existing DES model, previously used to asses the cost-effectiveness of long-acting injectable risperidone in Germany[14], was adapted to conduct the pharmaco-economic analysis of generic substitution of oral risperidone in Germany. The advantages of a DES model is that patient histories and patient heterogeneity can easily be taken into account when modelling future events, as opposed to cohort models (e.g. decision tree or markov model).[15,16] As schizophrenia is a complex, highly heterogeneous disease, DES is best suited to perform cost-effectiveness analyses for this indication.[17] Details of the model can be found elsewhere[15,18] however a brief description of the model will be provided with special attention given to the issue of compliance.

The model simulates individual patients suffering from schizophrenia over a period of five years and during this time a patient can be in two different health states: in a state of relapse or between relapses. Throughout the entire model horizon, the severity of a patient's symptoms is monitored by their Positive and Negative Syndrome Scale (PANSS). The PANSS score is the main element that influences the core model outcomes: Quality Adjusted Life Years (QALYs) and costs. There is a direct link between the PANSS score and quality of life, indeed Lenert *et al*. found the two variables to be negatively correlated[19] and quality of life is affected by the experience of side effects. An indirect link between the PANSS score and costs is established through the treatment setting chosen for a patient. Patients with a higher PANSS score have a higher level of dependency, leading to a higher probability of being treated in a more intensive and thus more costly treatment setting (e.g. day care departments or hospitals). In addition to treatment setting costs, total costs also consist of medication costs and psychiatrist visit costs.

Compliance with medication is modelled as a dichotomous variable; patients are compliant or non-compliant. The probability of being compliant depends on the treatment setting and a patient's health state. Firstly, it is modelled so that patients in a more intensive treatment setting (e.g. hospital) have a higher probability of being fully compliant[20,21] Secondly, based on expert opinion, it is modelled so that patients experiencing a relapse have a lower probability of being fully compliant than patients between relapses.

Compliance directly affects the PANSS score and the time between relapses. This implies that quality of life and costs are indirectly influenced by compliance. It is modelled so that among patients who are compliant, medication leads to a 20% PANSS score reduction during relapses, and a 5% reduction between relapses. [22-25] Patients who are non-compliant gain no benefits from medication; therefore they have no PANSS score reduction. If patients are compliant they are faced with a time between relapses ranging from 1.1 years (most severe patients) to 1.6 years (least severe patients). Patients who are non-compliant face a shorter time between relapses of between 0.29 to 0.43 years. [26-28]

To assess the impact of generic substitution on the costs and effects, the model was run with patients currently on branded risperidone that either switch to generic risperidone or stay on branded risperidone. Therefore the treatment arms are identical in every aspect except a difference in compliance and a difference in medication costs. It is modelled so that an individual patient who switches to generic risperidone will have a higher probability of becoming non-compliant than patients staying on branded risperidone. There is no published literature that quantifies the effect of generic substitution on compliance in schizophrenia, therefore several incremental probabilities of becoming non-compliant after generic substitution within a reasonable range (0%–10% with steps of 2.5%) have been explored. These differences are only applied to the three least intensive treatment settings, as it was expected that compliance differences would only hold in treatment settings with low supervision. These treatment settings are home-pp (patients visiting a private practice psychiatrist), home-ia (patients visiting *Institutsambulanz*, i.e outpatient clinics) and sheltered living. The other, more intensive treatment settings are day care, hospital and long term care institution. In a separate analysis, the incremental probability of becoming non-compliant after generic substitution is also applied to the latter treatment settings. Medication costs for the generic risperidone arm are assumed to be 40% lower compared to the branded price, which is currently €7.41.[29]

### Sensitivity Analysis

In a separate sensitivity analysis, the influence of different generic prices for risperidone is tested. A price reduction range of 20%–60% compared to the branded price is applied and incremental effects and costs are recorded.

Also, an analysis of the Expected Impact of Sample Information (EISI) on the probability of cost-effectiveness is conducted. The reason for this is that currently there is no study quantifying the compliance loss after generic substitution with risperidone in patients with schizophrenia, which makes the current analysis hypothetical to a certain extent. Therefore, a logical next step may be to conduct a study that quantifies the incremental probability of becoming non-compliant after generic substitution. The observed probability and corresponding uncertainty could be used to estimate the probability of branded risperidone being cost-effective.

Before conducting such a trial, the model can be used to assess the impact of changing the trial size (at different levels of expected compliance losses) on the probability to be cost-effective. Thus, this analysis can be used to estimate the number of patients required to show a certain probability of cost-effectiveness at various levels of compliance loss. This is performed in the model by choosing different values of reasonable compliance losses, in this case 6%, 6.5%, 7%, 7.5%, 8%, 9%, and 10%. Subsequently the probability of being cost-effective is calculated assuming the above compliance differences are observed in trials with 10, 20, 50, 100, 200 and 500 patients. Clearly, the uncertainty surrounding the compliance loss depends on the trial size; the more patients included in the trial, the lower the uncertainty. The corresponding probabilities of being cost-effective are estimated by performing 42 (7 compliance losses * 6 trial sizes) multivariate sensitivity analyses. It is expected that the lower the uncertainty surrounding the incremental probability of becoming non-compliant after generic substitution, the higher the probability that staying on branded risperidone will be cost-effective (if the base case is cost-effective).

## Results

Health and economic-related outcomes are presented in Table 1. Clearly, the only difference in the outcomes for branded risperidone and generic risperidone without compliance loss are the drug costs. This difference is explained by the 40% lower price of generic risperidone. There are no other modelled differences between these arms in terms of compliance, PANSS score reduction, side effects or other parameters that may influence QALYs or costs.

**Table 1:** Model outcomes for several compliance losses with generic substitution

	Branded Risp.	Generic Risp. 0%	Generic Risp. 2.5%	Generic Risp. 5%	Generic Risp. 7.5%	Generic Risp. 10%
**# Relapses**	3.98	3.98	3.99	4.01	4.02	4.04
**Total relapse time (yrs)**	2.05	2.05	2.06	2.07	2.07	2.08
						
**Average PANSS score**	62.9	62.9	63.0	63.2	63.4	63.5
						
**Time on risperidone (yrs)**	1.16	1.16	1.14	1.13	1.12	1.10
**% on risperidone after 1 yr**	60.7%	60.7%	59.3%	57.9%	56.5%	55.1%
						
**Total QALYs (disc)**	3.622	3.622	3.618	3.615	3.611	3.607
						
**Costs (discounted by component)**						
Home (pp)	€0	€0	€0	€0	€0	€0
Home (ia)	€254	€254	€255	€256	€257	€258
Sheltered Living	€7,120	€7,124	€7,136	€7,145	€7,159	€7,182
Day Care	€35,188	€35,229	€35,329	€35,479	€35,654	€35,758
Hospital	€37,124	€37,088	€37,440	€37,700	€37,971	€38,267
Long-term care institution	€958	€953	€966	€965	€972	€973
Psych Visits	€952	€952	€954	€955	€957	€959
Drug Costs	€8,012	€6,778	€6,772	€6,765	€6,760	€6,755
**Total Discounted Costs**	€89,607	€88,378	€88,850	€89,265	€89,730	€90,152
						
**ΔE**		0	0.004	0.007	0.011	0.015
**ΔC**		€1,230	€757	€343	-€123	-€544
**ICUR**		-	€196,243	€46,032	dominant	dominant

PANSS = positive and negative syndrome scale, QALYs = quality adjusted life years, ΔE = incremental effects of branded risperidone vs. generic risperidone, ΔC = incremental costs of branded risperidone vs. generic risperidone, ICUR = incremental cost utility ratio of branded risperidone vs. generic risperidone.

As could be expected, the larger the incremental probability of becoming non-compliant after generic substitution, the larger the average PANSS score is and the longer the total relapse time and the more treatment setting costs (particularly for day care and hospital) are incurred. Also, the average time on risperidone decreases as patients will switch treatment earlier due to lower efficacy. The QALYs also decrease as compliance losses increase, resulting from worsening symptom control (increase in PANSS scores). Thus, incremental effects of branded risperidone versus generic risperidone increase while incremental costs decrease as compliance losses are higher. Worsening symptom reduction implies lower quality of life and a higher probability of going to a more intensive and thus more costly treatment setting.

**Figure 1:**
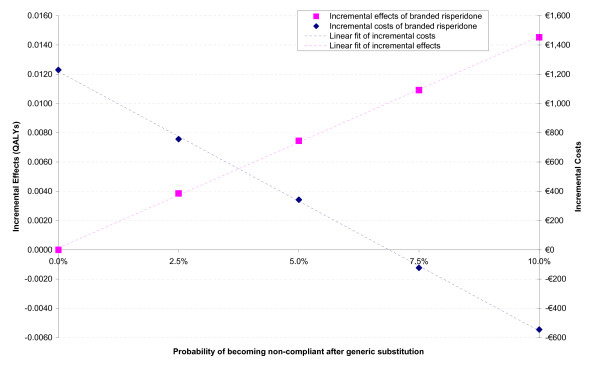
**Incremental effects and costs for various compliance differences (including linear lines)**.

With a 2.5%, 5%, 7.5% and 10% incremental probability of becoming non-compliant after switching to generic risperidone, incremental effects of branded risperidone compared to generic risperidone are 0.004, 0.007, 0.011 and 0.015 QALYs respectively. Incremental costs are €1,230, €757, €343, -€123 and -€544 respectively. Incremental effects and costs both have a linear relationship with the difference in compliance, as presented in Figure 1. With each percentage-point in compliance loss, incremental costs decrease by €177. Incremental effects on the other hand increase with 0.0014 QALYs for each additional percentage-point in compliance loss.

**Figure 2:**
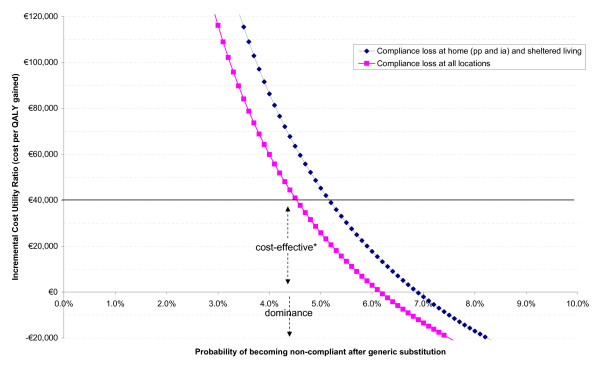
**Estimated Incremental Cost Utility Ratio at various compliance losses after generic substitution**. *At a willingness to pay of €40,000 per QALY gained.

Based on the linear lines representing the incremental effects and costs, the incremental cost utility ratios (ICUR) at various compliance differences can be determined by dividing the incremental costs by the corresponding incremental effects (see Figure 2). From Figure 2 it is clear that if the compliance loss after generic substitution is more than 5.2%, it may not be cost-effective (at a willingness to pay (WTP) of €40,000 per QALY gained) to switch a patient on branded risperidone to generic risperidone. Also, at a compliance loss of more than 6.9%, it is estimated that it may be cost-saving and lead to an increase in health benefits to not switch a patient on branded risperidone to generic risperidone.

If the compliance loss with generic risperidone is applied to all treatment settings (i.e. including the more intensive treatment settings), the more favourable the option to keep treating patients with branded risperidone. In this analysis, a patient who is treated in a more intensive treatment setting also has a higher probability of becoming non-compliant after generic substitution. The minimal incremental probability of becoming non-compliant after generic substitution needs to be 4.5% in order for branded risperidone to be cost-effective compared to switching a patient to generic risperidone at a WTP threshold of €40,000 per QALY gained. If the incremental probability of becoming non-compliant is more than 6.2%, branded risperidone is estimated to generate cost-savings and health-benefits compared to switching a patient to generic risperidone (Figure 2).

### Sensitivity Analysis

Table 2 shows the minimal compliance losses at which staying on branded risperidone is cost-effective and is the dominant treatment option respectively compared to generic substitution at various prices for generic risperidone. Note that for this analysis, the incremental probability of becoming non-compliant after generic substitution is only applied to the three least intensive treatment settings. As can be expected, the lower the price of generic risperidone, the higher the incremental probability of non-compliance must be in order for branded risperidone to be cost-effective or dominant. A larger difference in medication costs must be bridged by larger treatment setting cost-savings (and larger health-benefits) which can only be achieved by larger compliance differences.

**Table 2:** Compliance loss thresholds for various price levels of generic risperidone at which staying on branded risperidone is cost-effective and dominant

**Price reduction for generic risperidone (compared to branded price)**	Min. compl. loss^† ^for cost-effectiveness*	**Min. compl. loss^† ^for dominance**
20%	2.8%	3.7%
30%	4.1%	5.5%
40%	5.2%	6.9%
50%	6.7%	8.8%
60%	7.9%	10.5%

† Only applied to the three least intensive treatment settings

*At a willingness to pay of €40,000 per QALY gained

**Figure 3:**
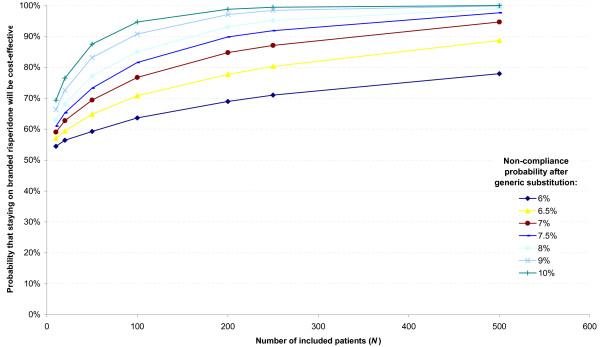
**Probability that staying on branded risperidone will be cost-effective (at a WTP of €40,000 per QALY gained) compared to generic substitution (at various levels of probability of becoming non-compliant after generic substitution and with various number of patients included)**.

The results of the EISI analysis are presented in Figure 3 which shows that the larger the size of the trial and the larger the incremental probability that switching to generic risperidone results in non-compliance, the higher the probability that branded risperidone will be cost-effective at a WTP of €40,000 per QALY gained. For example Figure 3 shows that for a trial including 200 patients that finds a 7% probability of becoming non-compliant after generic substitution, the expected probability of branded risperidone being cost-effective is 85% at a WTP of €40,000 per QALY gained. Also, it should be noted that there is a declining marginal benefit of adding one more patient. In other words, adding one patient to a group of 200 does not increase certainty as much as adding one patient to a group of 10 people for instance.

## Discussion

The pharmaco-economic DES model predicts that generic substitution of risperidone in patients suffering from schizophrenia in Germany may not be cost-effective/cost-saving if it involves a loss in compliance. Patients who become non-compliant after generic substitution have less symptom control which increases the probability of being treated in a more intensive and thus costly facility (e.g. hospital). The costs incurred in such treatment settings (partially) counteract the drug cost savings resulting from generic substitution. Also, less symptom control in patients suffering from schizophrenia negatively affects the quality of life. The balance between drug-cost savings on one hand and additional treatment setting costs and worse quality of life on the other hand depends on the degree of the compliance loss and the difference between the branded and the generic price. The smaller the price difference, the smaller the compliances loss needs to be to offset the drug cost-savings.

Kluznik *et al*. also acknowledge that generic substitution in schizophrenia may not be cost-saving due to decreased symptom control.[30] However in their study they considered clozapine. In this study, lower symptom control after generic substitution was caused by a clinical difference between both compounds instead of less compliance with generic clozapine. In fact, both tablets looked identical to avoid patients finding out they were switched to a generic alternative.

To the best of the authors' knowledge, no study has yet been conducted that quantifies the incremental probability to become non-compliant after generic substitution in patients suffering from schizophrenia. If such a trial would be conducted, the validity of the present compliance loss assumptions can be tested according to the trial results. Also, an EISI analysis was conducted, providing a tool to relate the findings of this trial (and corresponding uncertainty) to the probability that staying on branded risperidone would be cost-effective compared to generic substitution.

For a psychiatrist it is difficult to accurately estimate the additional risk of non-compliance after generic substitution for a particular patient. As reported by Fleischhacker *et al*., there are several factors influencing compliance, therefore also affecting the additional risk of non-compliance after generic substitution.[8] Careful consideration of these factors may help a psychiatrist in assessing the likelihood that a particular patient may end up being non-compliant after generic substitution. If a patient showed more symptoms or ended up in a relapse after a previous substitution, it is probably not cost-effective, nor in the interest of the patient to substitute his medication with a generic alternative. Also, if a patient has a negative attitude towards changes in general or suffers from paranoia or delusions, generic substitution is more likely to result in non-compliance. On the other hand it is not unlikely that paranoid or delusional patients will already be non-compliant on the current treatment. Patients having a supportive environment are more likely to be compliant than patients living alone.[31] Similarly, one may expect that generic substitution is less likely to result in non-compliance for patients living in a supportive environment. On the other hand it is essential that a patient's environment has a positive attitude towards treatment changes and is able to understand and explain the rationale. This may prove to be difficult in cases for which a patient is stable on a current regimen. A cornerstone of treatment compliance is the therapeutic relationship between the clinician and the patient.[32] A structured nature of the therapy is a signal to the patient that his therapy is of importance.[8] A stable therapy structure may be impaired by changing a patient's medication.

The current DES model is an adaptation of previous models[14,15,18] and distinguishes itself from the other models in that it specifically addresses the issue of compliance loss with generic substitution of risperidone in Germany. DES is a suitable and flexible technique for modelling heterogeneous and complex diseases with numerous interdependencies such as schizophrenia. Model inputs and design were based on data from the literature whenever possible. Nevertheless, due to the lack of long-term data, it was unavoidable to make a few assumptions which have been substantiated by previously consulted experts. To validate the model, outputs were compared with published sources. Schulenburg *et al*. reported direct schizophrenia cost of €14,204 per patient in Germany in 1996.[33] If we adjust this figure to 2007 costs using the German price consumer index[34], this would give an estimated figure of approximately €16,800. The annual costs in the DES model (€18,000) are very close to this figure. The model estimates that patients spend 2.1 out of 5 years in relapse, which is very close to the estimate of 40% reported by Mason *et al*.[35] Hence, the model results seem to be in line with published estimates.

## Conclusion

It is estimated that at a WTP threshold of €40,000 per QALY gained, it is cost-effective to keep a patient with schizophrenia in Germany on branded risperidone instead of switching him/her to generic risperidone (assuming a 40% reduction in medication costs), if the incremental probability that he/she will become non-compliant after generic substitution is more than 5.2%.

## Competing interests

Maarten Treur and Bart Heeg are employed by, and Ben van Hout is partner of Pharmerit. Pharmerit holds consultancy contracts with Janssen-Cilag. Annette Schmeding is employed by Janssen-Cilag GmbH. Professor Möller has received research grants/support from, serves as a consultant or is on the advisory board for, or is a member of the speaker bureau for the following companies: AstraZeneca, Bristol-Myers Squibb, Eli Lilly, Eisai, GlaxoSmithKline, Janssen Cilag, Lundbeck, Merck, Novartis, Organon, Pfizer, Sanofi Aventis, Sepracor, Servier, Wyeth.

## Authors' contributions

MT was involved in developing the model, carried out the analyses and drafted the manuscript. BH developed the model and helped to draft the manuscript. HJM assisted in developing the model and design of the analyses, and was involved in revising the manuscript. AS assisted in developing the model and helped drafting the manuscript. BVH developed the model and was involved in revising the manuscript. All authors read and approved the final manuscript.

## Pre-publication history

The pre-publication history for this paper can be accessed here:


